# Lessons learned from Korea: COVID-19 pandemic

**DOI:** 10.1017/ice.2020.104

**Published:** 2020-04-03

**Authors:** Hazhir Moradi, Atefeh Vaezi

**Affiliations:** 1 School of Medicine, Isfahan University of Medical Sciences, Isfahan, Iran; 2 Department of Community and Family Medicine, School of Medicine, Isfahan University of Medical Sciences, Isfahan, Iran

*To the Editor*—The World Health Organization (WHO) declared COVID-19 a pandemic on March 11, pointing to >118,000 cases of coronavirus pneumonia worldwide.^1^ On the last day of 2019, China reported some cases of pneumonia with unknown etiology in Wuhan. Approximately 7 days later, gene sequencing revealed that the etiologic agent was a coronavirus, which was subsequently named SARS-Cov-2.^2^ As of March 18, 2020, >218,000 infected patients and 8,900 deaths had been reported, and the virus had reached 173 countries.^3^

To control an outbreak, every country needs to have preparedness, alert, and response plans.^4^ Preparedness comprises activities that began before the crisis; its goal is to create infrastructure and to empower public health workers. Alert plans comprise activities conducted to detect and verify the outbreak, and response activities during the crisis focus on controlling the problem.^5^

The first individual with COVID-19 in Korea was detected on January 20, 2020.^6^ Today, 60 days after the first case, statistics show that the peak of infection has passed. A total of 8,413 cases have been confirmed, and the number of new cases has reached <100 for the fourth day in a row.^3^ In this study, we describe the outbreak response and preparedness activities that Korea implemented to control the COVID-19 epidemic.

The outbreak alert system in Korea has 4 levels: (1) attention to the outbreak, in which the government began to monitor and prepare; (2) caution when the outbreak entered the country and the government operates cooperation system; (3) alerts regarding the spread of infection to other areas and initiation of the response system; and (4) mobilization of a nationwide response system as the outbreak spread and became severe.^7^

Four days after the notification of new cases in China,^8^ while the source was not yet clear, Korea started screening and implemented a quarantine plan at the airports. Those who had visited Wuhan in the previous 14 days were required to complete a health questionnaire and to self-quarantine for 14 days. If fever or respiratory symptoms appeared, they were required to call the Korea Centers for Disease Control and Prevention (KCDC).^3^

On January 20, the first case of COVID-19 pneumonia, which was detected in the airport screening station, was confirmed,^6^ resulting in the elevation of the infection alert level from blue (attention) to yellow (caution). In-depth epidemiological studies were conducted, and all contacts were followed for 14 days. These individuals were isolated and tested if any symptoms appeared, and all of the places where the case patients had gone (eg, hotels, markets, and health facilities) were disinfected.^3^ On February 21, when epidemiologic studies revealed 2 main sources of transmission, those places were defined as “special care zones” where a specialized team focused on controlling transmission,^9^ and the alert level was elevated to the highest (severe).^10^

Rapid diagnosis and widespread testing were other areas of focus in Korea. The proportion of confirmed to suspected cases varied from 0.5 in the initial days to 3.9 in the peak days. Early detection helped Korea eliminate the infection from the community and restrict it to health facilities, which is an essential aspect of outbreak response. Also, research teams started their work in the very early days to develop rapid tests, treatments, and vaccines. From January 31 onward, the 6-hour test was distributed in some health facilities, and from February 7 onward, all health facilities all around the country had this test.^10^

Moreover, the KCDC started reporting the situation from January 20 onward to provide accurate and real-time data. These reports included the number of confirmed cases and patients under investigation, history of confirmed cases, and prevention advice for the public. The number of the KCDC call center has been mentioned in almost every report, and Koreans were asked not to travel to China and Wuhan, to avoid public outdoor activities, to cough or sneeze safely, and to wear masks when visiting a health center. Besides, the guideline for management and screening get updated whenever needed; travel to Wuhan which was in the definition of suspected cases where changed to travel to china, and finally omitted.^10^

Altogether, the main goal of outbreak response in Korea was prevention of entrance of COVID-19 and at the same time, inhibition of the spread of the virus throughout the country. These goals were achieved through 3 main strategies: (1) containment and mitigation based on outbreak situation; (2) Risk communication to attract community participation; and (3) science-based and fact-driven actions.
